# Tuberculosis control in the face of emerging infectious diseases: Highlights from the 7th South African Tuberculosis Conference

**DOI:** 10.1002/puh2.63

**Published:** 2023-06-07

**Authors:** Shamsuddeen Yusuf Ma'aruf, Tayla Smith, Su‐Mari du Plessis, Zimvo Obasa

**Affiliations:** ^1^ NRF/DSI Centre of Excellence for Biomedical Tuberculosis Research; South African Medical Research Council Centre for Tuberculosis Research; Division of Molecular Biology and Human Genetics, Faculty of Medicine and Health Sciences Stellenbosch University Cape Town South Africa; ^2^ School of Medical Laboratory Science Usmanu Danfodiyo University Sokoto Nigeria

**Keywords:** emerging infectious diseases, health policy, stakeholders, tuberculosis, vaccine

## Abstract

Tuberculosis (TB) remains a disease of global concern caused by the pathogen *Mycobacterium tuberculosis*. In the face of emerging infectious diseases, such as COVID‐19, dengue fever, Lassa fever, and Ebola, attention to the control of TB is losing its place as a priority in public and global health initiatives. Recently, after a hiatus enforced by the COVID‐19 pandemic, scientists, public health experts, and other stakeholders met in Durban, South Africa (SA) for the 7th South Africa Tuberculosis Conference to share, discuss, and recommend strategies to regain TB control. In this paper, we summarized and captured key plenary sessions and presentations by scientists and other stakeholders at the conference, which focused on various themes: pathogenesis of TB—pathogen and host, drugs/vaccine/diagnostics, implementation/health systems, and social and community aspects of TB. The current policy and effort to control TB have declined in recent times. We suggested a critical and inclusive engagement of funders, researchers, policymakers, and the civil society toward regaining TB control. Additional investigation into this paper may make it easier to identify control strategies to help realize the End TB Strategy.

## INTRODUCTION

Tuberculosis (TB) caused by the pathogen *Mycobacterium tuberculosis* (*Mtb*) remains a disease of global concern [1]. Attention on TB by researchers, healthcare workers, and stakeholders in tackling TB both globally (international programs) and at the ground level (local implementation) has shifted attention away from TB and toward emerging infectious diseases (EIDs), such as COVID‐19, Lassa fever, Ebola, and Dengue fever.

**FIGURE 1:**
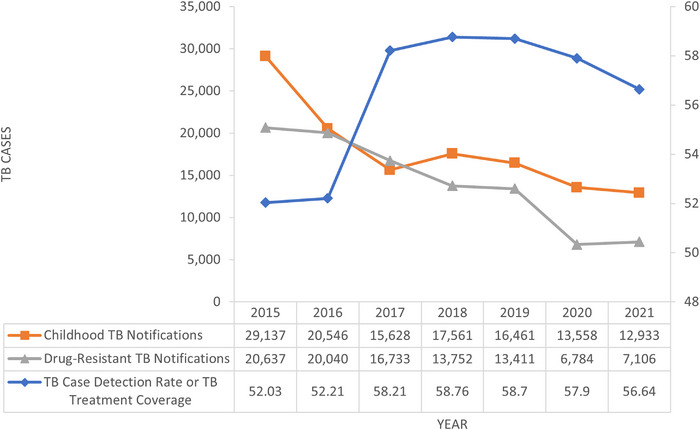
Tuberculosis (TB) case notification trends in South Africa, 2015–2021 [5]

Many countries have seen changes in their TB situations. Nigeria has witnessed a 15% increase in TB cases, which could be due to a limited control policy or the impact of the COVID‐19 pandemic [2]. India and South Africa (SA) have achieved certain milestones in their TB control, such as decreased death rate and increased number of diagnoses [3], but there are still challenges that need to be addressed. In SA, as noted by the Deputy Minister of Health, Dr. Sibongiseni Dhlomo, between March and June 2020, monthly drug‐resistant (DR) TB diagnosis notifications fell by more than 50% [3, 4] (Figure 1). The reason for the disruption could be due to the reallocation of resources, decreased TB services, and multi‐complex supply chain and logistics shift. During the 7 weeks preceding the lockdown in June 2020, there was an average of 49,109 TB tests per week. During the lockdown, there was a drop in the average of 24,620 tests per week. The weekly average of microbiologically proven TB cases decreased by 33% over the same time period, from 3707 to 2465 cases per week on average [3].

Local and international stakeholders registered their interest at the first in‐person TB conference since 2018 to express their concern about decreasing efforts to tackle TB in SA and globally. The conference was held over 4 days at the International Conference Centre (ICC) in Durban, SA from September 13 to September 16, 2022. It comprises four morning plenary sessions, as well as four track‐breakout sessions, namely, the pathogenesis of TB—pathogen and host, drugs/vaccine/diagnostics, implementation/health systems, social and community aspects of TB, and other satellite sessions. The goal of the 7th South African TB Conference was to strengthen TB control in‐line with the WHO End TB recommendation. Field workers, 135 health managers, and scientists were all present at the conference. About 40 research works were presented orally with about 123 poster presentations under various tracks [4].

This article highlights the potential policies and scientific outcomes of the discussions during the conference. This article is subdivided into themes according to the tracks of the conference.

### Revisiting the pathogenesis of TB

Most TB cases have pulmonary involvement and are often brought about by inhaling aerosolized *Mtb* [6]. Inhalation of *Mtb* leads to one of four possible outcomes: immediate clearance of the organism, latent infection, the onset of active disease (primary disease), and active disease many years later (reactivation disease) [7]. The likelihood of transmission increases with the presence of bacterial burden in the source patient, cavitary or upper lung zone disease on chest radiograph, laryngeal disease in the source patient, amount and severity of coughing in the source patient, duration of exposure of the contact, proximity of the contact to the source patient, crowded conditions and poor ventilation in the room, and delays in diagnosis and/or effective treatment of the source patient [8]. Understanding the pathogenesis of TB has gone through various eras. In the early era of TB, granuloma was identified as an extension of prolonged TB infection. Granulomas in postprimary TB form as a reaction to retained caseous pneumonia [9].

### Health system of South Africa and TB epidemiology

SA health system is managed by the provincial government across the nine provinces: Eastern Cape, Free State, Gauteng, KwaZulu‐Natal, Limpopo, Mpumalanga, Northern Cape, North West, and Western Cape Province. There were approximately 57.9 million people living in SA in 2018, with a population density of 47.3 persons per km^2^ and an estimated 15.4% coverage of medical schemes/health insurance as seen in Table 1.

**TABLE 1:** The estimated coverage of maternal and reproductive health, infectious disease control, noncommunicable diseases, and other healthcare services in South Africa

Provinces	Estimates (%) [10]
Eastern Cape	9.8
Free State	13.5
Gauteng	24.6
KwaZulu‐Natal	11.2
Limpopo	7.2
Mpumalanga	12.5
Northern Cape	15.1
North West	11.9
Western Cape	20.1

In relation to universal health coverage and health‐related Sustainable Development Goals (SDGs), the country has achieved remarkable success across various health sectors, such as in maternal and reproductive health, infectious disease control, noncommunicable diseases, other healthcare services, and capacity development [10]. SA has 52 health districts across the country and nearly 3500 public health clinics, which provide a variety of services, including the diagnosis and treatment of TB and human immunodeficiency virus (HIV). They also serve as the “first line” of the country's healthcare system [11].

The World Health Organization (WHO) estimates that over 110,000 people with TB in SA lost their lives between 2020 and 2021 [2]. In 2016, SA adopted the TB 90–90–90 goals. By December 2020, this approach sought to screen 90% of high‐risk and vulnerable groups for TB, diagnose and treat 90% of those who have the disease, and successfully treat 90% of patients with drug‐sensitive TB (TB DS) or 70% of patients with DR TB. In the National Strategic Plan (NSP) for HIV, TB, and sexually transmitted infections 2017–2022, these goals were changed to 90–100–90 [10]. Currently, majority of TB diagnoses are made casually using the WHO four‐symptom indicators, with sputum being screened using the Xpert Cepheid MTB/RIF System [11].

SA has a strong research infrastructure that enables it to conduct clinical trials with good data management. It has made significant contributions to numerous studies that have impacted HIV and TB preventions and treatments around the world [3]. Unfortunately, researchers and other data producers are hesitant to disclose their data unless they are certain that it is of high quality and is reliable, and that it is produced and used in accordance with the principles and objectives of their organizations [12]. In addition, many studies are externally funded, which require the permission from the funders to have study protocols that bind them. These are some of the reasons for data sharing hesitancy.

The WHO has made available TB country profile and other reports online and made TB report more seamless using the free WHO TB Report mobile app [1]. There are various TB data management software systems, such as the national electronic TB register (ETR.Net) database for TB DS, the electronic DR‐TB register (EDRWeb) for DR‐TB, and the Three Integrated Electronic Registers (Tier.Net). Professor Norbert Ndjeka stated that only a select group of stakeholders have access to certain parts of the platforms. This was debated at the conference, and it was agreed to be made available to other stakeholders to improve TB patient outcomes and management. Furthermore, data evaluation or auditing should be encouraged, and various TB management platforms should be integrated or centralized [13].

### Social and community aspects of TB control

The importance of collective efforts in TB control has been emphasized by Steve Letsike of the SANAC National Civil Society Forum when he stated that, “TB is everyone's problem. A lot more can be done if we work together and not in silos.” [4]. This has been emphasized by the following developments. Recently, there are some evidence that there is an associated risk among smokers with prior TB developing severe emphysema and a higher prevalence of bronchiectasis [14]. The Deputy Minister of Health of SA stated in his address that the country reports around 300,000 cases and 25,000 deaths due to TB annually. He added that during the COVID‐19 pandemic, global TB cases also increased. He reported that the National Health Department has crafted a Control of Tobacco Products and Electronic Delivery Systems Bill, which, if passed, will help assist in controlling TB and tobacco [4]. Hence, the need to control tobacco would involve both policymakers and researchers. Institutions such as the Stellenbosch University and other institutions recently declared a provisional position urging its researchers not to apply for or accept research funding and engagement from the tobacco industry [15].

Similar to those with HIV/AIDS and leprosy, TB patients also face persistent stigma [16]. A study in India, which is one of the countries with high TB burden, reported that 51.2% of respondents experienced some form of stigma associated with the disease [17]. Research shows how stigma can have a significant influence on individuals and communities, including delays in seeking medical attention and adherence to therapy [18]. Significant changes in the practice of TB care have been observed in recent years by notable members of the TB community, including the Stop TB Partnership, Treatment Action Group, TB PROOF, Global Coalition of TB Activists, and the KNCV (Koninklijke Nederlandse Centrale Vereniging or the Royal Dutch Central Association) TB Foundation. Terminology that supports unfair stereotypes of people with TB is being removed from the lexicons of clinicians and scientists [19]. According to health activist Phumeza Tisile, who was diagnosed with susceptible TB, while all along she had extensively DR TB (XDR‐TB), a situation that left her deaf in both ears, said, “TB cases are not subjects and TB suspects are not criminals.” In her presentation, she added that the uses of colored cards and the phrase “missing cases” are a potential source of stigmatization [20]. It was agreed that the use of phrases or indicators to describe TB patients needs attention to avoid stigmatization.

Efforts to respond to TB need to involve the communities affected [21]. Resources need to be deployed to different communities such as those collaborating with traditional leaders. There is a need to broaden the TB advocates community through increase in funds [22]. As discussed in a coffee corner event organized by the Working Group for New TB Vaccine, one of the participants suggested that conducting conferences in low‐ and middle‐income countries, which are facing the major burden of the disease, should be prioritized [23]. This suggestion provides them with opportunities to access more funding, particularly toward research and development. It also allows TB survivors and advocacy groups to get involved and share their perspectives and experiences with researchers to ensure patient‐centered research studies.

### Diagnostics, drugs, and vaccine for TB

There could be an additional 400,000 TB‐related fatalities in 2020 globally because of a 25%–50% drop in the number of patients detected and treated for TB according to one study presented [3]. A survey, which covered 106 countries, indicated that COVID‐19 impaired TB services in 78% of these countries in 2020, with substantial or very high disruptions occurring in 17% [3]. For example, SA has observed a decline in TB notifications in various provinces [3, 24]. This remains above the WHO crisis level at about 200 per 100,000 population [25]. This might not necessarily be due to efforts to control TB but attributed to the impact of the COVID‐19 pandemic. It is opposite to other high TB cases in countries like Nigeria, Uganda, India, and Zambia, where these countries have witnessed an increase in number. COVID‐19 has reversed the gains in SA, which have started to be observed [26].

In the context of the pandemic and as the world approaches 2030, there are five risk factors in the achievement of SDGs indicators associated with TB incidence. These include HIV and undernourishment, in contrast to other common risk factors, such as alcohol, smoking, and diabetes [27]. The increasing demand for resources and attention to COVID‐19 patients is placing added strain on healthcare systems that would traditionally commit to TB and HIV diagnosis and treatment programs [28]. Countries have started to address this such as India, which has made progress in reducing TB infections by recapacitating diagnostics in‐line with COVID‐19 testing over the last 2 years [29]. Other countries need to establish similar plans and strategies to rework diagnostic capacities in facing new health challenges such as other EIDs.

Innovative digital solutions, such as the use of Short Message Service, which was rolled out in SA in 2022 [30], apps, phone calls, informing patients about their results or sending treatment reminders, and referrals to community health centers, can be effective in managing TB numbers or patient follow‐ups. Creating public health data centers could also provide an effective support to TB programs [22]. GeneXpert has proven to be effective in the diagnoses of TB, but this technology shows limitations as it cannot be deployed to the field or to the remote areas [22]. Several solutions are now in the pipeline to address the gap or limitations. These include TBCheck [31], Innovate TB [32], the use of Geographical Information Systems to find missing TB cases, a non‐sputum antibody detection point‐of‐care testing TB diagnostic device [33], digital chest radiography, and lipoarabinomannan testing. Advanced diagnostic techniques should be accepted and established to complement the release of new drugs such as in the detection of XDR‐TB through the use of whole genome sequencing (WGS) [34]; however, this might be inaccessible in some communities. This is addressed by a new GeneXpert for XDR‐TB, which could complement the limitation of WGS.

The WHO has urged scientists and researchers to contribute to the scaling up of novel and shorter regimen drugs for TB and the implementation of TB preventive therapy such as the 3HP [35]. The 3HP includes rifapentine and isoniazid taken for 3 months to improve TB prevention and reduce transmission. Recently, the WHO published a new 4‐month regimen for drug‐susceptible‐TB (DS‐TB). This takes into consideration the pharmacokinetics of new TB drugs as well as the complexities of treating people living with HIV (PLHIV) [36]. Nanomedicine is also gaining momentum in TB treatment [37, 38].

Within the realm of TB vaccine development, scientists at the conference presented their attempts on various strategies of improving the current vaccine or identifying new candidates. The current and old Bacillus Calmette‐Guérin (BCG) vaccine is more effective in teenagers and only offers 33% protection in adults [39]. The administration of BCG through a different route could potentiate the effect such as parenteral routes [40]. Similarly, Professor Keertan Dheda of the University of Cape Town presented the use of a pulmonary TB vaccine model to assess if nasally administered or inhaled BCG showed improved efficacy compared to normal administration of BCG vaccine by injection [39]. Dr. Munyaradzi Musvosvi presented their assessment of antigens bound to the T‐cell receptors during TB infections to identify potential antigens for inclusion in TB vaccine [41]. Dr. Marakalala Jackson mentioned that during granuloma development, host proteins associated with tissue destruction could also be recreated as a target for therapeutics [42].

The currently available tools for TB control might not be sufficient in reaching the 2030 target (a world free of TB) even by 2035 [1] if the efforts continue at the current pace. Scientists need to continue understanding why it is taking long to develop a new TB vaccine; this situation is already over 100 years after the introduction of the BCG vaccine. The lack of effective vaccines limits our ability to control the TB epidemic [43].

The current vaccines in the pipeline at various trial phases include M72 + ASO1, BCG‐ZIMP1, VPM1002, MVAC, GamTBVac, ID93, MIP, MVA Multiphasic vac. Transgene, CysVac2/Ad, H107, H56:IC31, TB/Flu01L, TB/Flu04L, and DAR‐901. VPM1002 and M72 + ASO1 have shown to be promising against TB disease [44], but as Dr. Luabeya pointed out, “Why are TB vaccines taking long when compared to COVID‐19?” [43]. Considering the factors that impacted on the COVID‐19 vaccine strategy, such as availability of funds, robust research, and global interest, TB vaccines should be introduced at a similar fast and effective rate, which might accelerate efforts to meet the target by 2035 [45].

## CONCLUSION

As the COVID‐19 pandemic efforts continue, TB pandemic control should not be allowed to decline. The deployment of resources for TB control from the top level down to the target audience should be discouraged because it ultimately dilutes the resources required for TB control. It is necessary to develop and adopt diagnostic tools, such as point‐of‐care testing for biomarker signatures or even mobile chest X‐rays. To close the gaps in accessing high‐quality medical care, innovative creative solutions should be produced, and data should always be made public because doing so will increase transparency. Like COVID‐19, vaccines should be distributed and validated at an efficient pace. Finally, TB control efforts must be inclusive and should engage the health departments, relevant ministries, the civil society, including funders, advocacy organizations, pharmaceutical companies, and the academia. If it can be done to control COVID‐19, the same thing can be done for TB.

## AUTHOR CONTRIBUTIONS

Shamsuddeen Yusuf Ma'aruf conceived the idea of the paper. Shamsuddeen Yusuf Ma'aruf and Tayla Smith wrote the first draft. Su‐Mari du Plesis and Zimvo Obasa reviewed and completed the final draft. All authors contributed intellectual content to the paper. All authors approved the last version of the paper.

## CONFLICT OF INTEREST

The authors declare that they have no conflict of interest.

## Data Availability

All relevant data sources are cited in this article.
